# Lung injury caused by aspiration of organophosphorus insecticide and gastric contents in pigs

**DOI:** 10.1080/15563650.2022.2028803

**Published:** 2022-02-11

**Authors:** Elspeth J. Hulse, Richard E. Clutton, Gordon Drummond, Adrian P. Thompson, Edwin J. R. van Beek, Sionagh H. Smith, Michael Eddleston

**Affiliations:** a Pharmacology, Toxicology, and Therapeutics Department, University/BHF Centre for Cardiovascular Science, University of Edinburgh, Edinburgh, UK; b Wellcome Trust Critical Care Laboratory for Large Animals, University of Edinburgh, Edinburgh, UK; c Anaesthesia, Critical Care and Pain Medicine department, Division of Health Sciences, University of Edinburgh, Edinburgh, UK; d Edinburgh Imaging, Queen’s Medical Research Institute, University of Edinburgh, Edinburgh, UK; e Easter Bush Pathology, Royal (Dick) School of Veterinary Studies and Roslin Institute, University of Edinburgh, Edinburgh, UK

**Keywords:** Organophosphorus insecticide, self-poisoning, aspiration, lung injury, pig

## Abstract

**Introduction:**

Patients who require mechanical ventilation after self-poisoning with ingested organophosphorus (OP) insecticides often die. Aspiration of stomach contents may contribute to lung injury and lethality. This study was designed to assess the severity of direct and indirect pulmonary injury created by pulmonary instillation of mixtures of OP insecticide, solvent (Solv) and porcine gastric juice (GJ) compared to controls.

**Methods:**

Terminally anaesthetised minipigs (groups *n* = 5) were exposed to sham bronchoscopy or given mixtures (0.5 mL/kg) of: saline, GJ, OP insecticide and GJ (OP + GJ), or Solv and GJ (Solv + GJ), placed into the right lung, and monitored for 48 h. Lung injury was assessed through analysis of bronchoalveolar lavage fluid (BALF), computed tomography and histopathology.

**Results:**

OP + GJ created a direct lung injury consisting of neutrophil infiltration, oedema and haemorrhage, as well as indirect injury to the other lung. OP + GJ directly-injured lung parenchyma had increased concentrations of BALF protein, albumin, IL-6, IL-8 and C-reactive protein (CRP) at 24 h (*p* < 0.05), and BALF protein, albumin and CRP at 48 h (*p* < 0.05), when compared with controls. Aspiration of GJ produced similar direct effects to OP + GJ but less indirect lung injury. Lung injury was less severe after Solv + GJ, for combined lung histopathology scores (vs. OP + GJ, *p* < 0.05) and for the proportion of directly-injured lung that was poorly/non-aerated at 48 h.

**Conclusion:**

Pulmonary instillation of OP + GJ created more lung damage than controls or Solv + GJ. In patients with severe OP insecticide poisoning and reduced consciousness, early airway protection is likely to reduce pulmonary damage.

## Introduction

Ingestion of pesticides remains one of the most common causes of suicide worldwide, particularly in rural Asia, with up to 150,000 annual deaths, mainly from organophosphorus (OP) pesticides [1–3]. OP compounds inhibit acetylcholinesterase (AChE), causing an acute cholinergic crisis and respiratory failure through effects on the central, autonomic and peripheral nervous systems [4]. In severe poisoning, unconsciousness is accompanied by signs of muscarinic receptor stimulation, i.e., bronchorrhea, bronchospasm and vomiting. At the same time, many patients experience signs of nicotinic receptor stimulation, including neuromuscular weakness and paralysis, increasing the likelihood of gastric contents aspiration [5,6]. A further risk factor for aspiration after presentation to hospital is the common and hazardous practice of gastric lavage, often prior to tracheal intubation and ventilation [6,7]. Here, a balance must be struck between trying to quickly remove the toxin from the stomach versus a time delay for airway protection, which may not be readily available in resource poor settings.

The case fatality for OP insecticide ingestion is approximately 10%, depending on the individual insecticide, the dose and time to medical care [8]. In moderate–severely poisoned patients, atropine reverses muscarinic toxicity while mechanical ventilation and perhaps oximes address nicotinic paralysis, increasing the likelihood of patient recovery [9–11]. Approximately 25% of patients are intubated/ventilated for respiratory failure, of whom 23–50% die [12–14]. For spraying efficiency, the insecticide is mixed with a solvent, such as xylene or cyclohexanone. We hypothesised that some deaths in ventilated patients result from acute lung injury caused by aspiration of gastric contents and that solvents worsen this lung injury [15].

Aspiration of acidic gastric contents causes lung parenchymal inflammation and oedema within hours [16]; however, the effects of aspiration of mixtures of OP insecticide along with gastric contents are unknown. To investigate this question, we developed a large animal study, over a clinically relevant time scale to describe, and where possible measure, the severity of bilateral lung injury created by instillation of mixtures of OP insecticide, gastric juice and a common OP insecticide solvent into one lung.

## Methods

The basic minipig model was established as previously reported [15,17,18]. Further details on study methodology and analysis are presented in the online supplement.

### Animals, study groups and anaesthesia

The study was approved by the Institutional Ethical Review Committee. Twenty-six female Gottingen minipigs, mean (±SD) weight 28 (±2) kg, were housed/treated as per Home Office (UK) guidelines. Pigs were terminally anaesthetised and intubated with a TCB Univent endotracheal tube (Fuji systems, Tokyo) to allow bronchial blockage of one lung during instillation of pulmonary mixtures to the contralateral lung [18]. Two pigs were studied simultaneously over 48 h and lungs were ventilated using a mixture of oxygen and medical air (F_i_O_2_ 0.5) with a tidal volume of 6–8 mL/kg delivered at 15–25 breaths per min. Peak inspiratory pressure (Ppeak) was limited to < 25 cm H_2_O, positive end expiratory pressure (PEEP) was set at 5 cm H_2_O and end-tidal CO_2_ maintained (mean ± SD) at 5.5 ± 0.8 kPa. Maintenance of anaesthesia was achieved with intravenous (IV) propofol (mean 11 (range 7–15) mg/kg/h) and fentanyl (mean 5 (2–12) mcg/kg/h) supplemented as required with IV midazolam.

**Figure 1.**
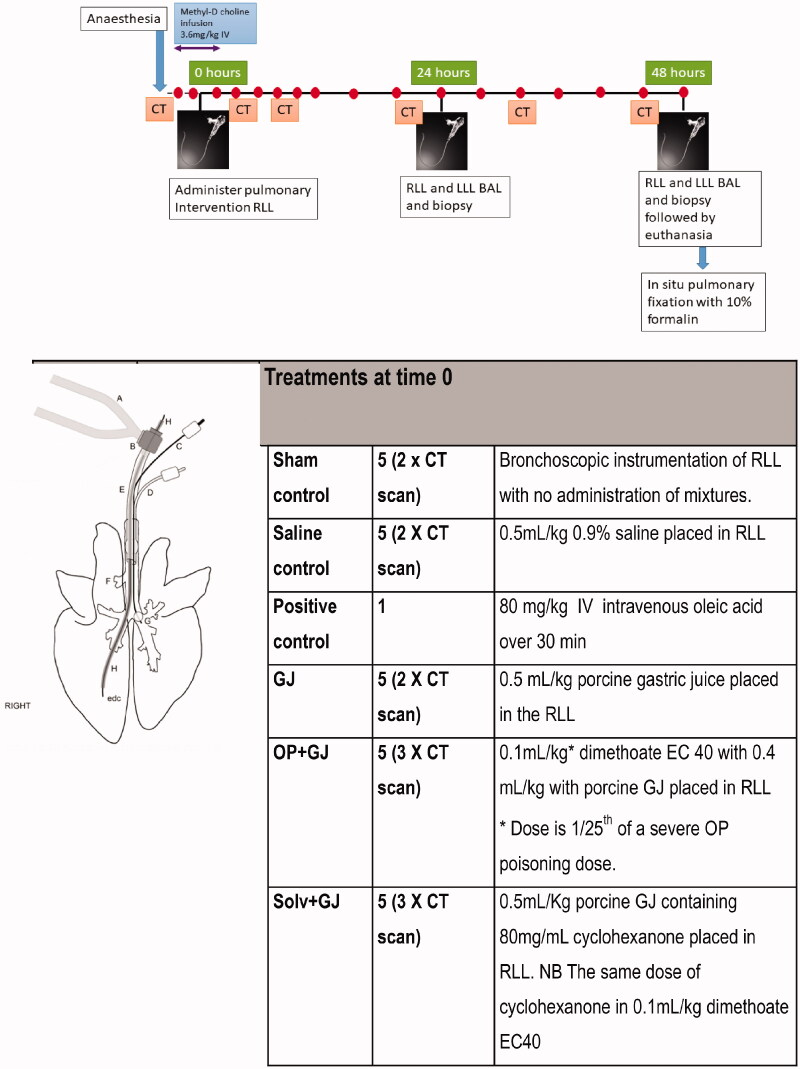
Minipig pulmonary aspiration protocol. Each minipig was anaesthetised approximately 2 h before time = 0 to allow time for surgical placement of arterial, central venous and urinary catheters. The pulmonary surfactant tracer isotope (methyl D9 choline chloride 3.6mg/kg) was infused IV from –30 min for 3h (data not shown). The dots denote sampling points for arterial and venous blood, +/- urine (data not shown). CT of lungs occurred at –30 min, 4, 8, 24, 32 and 47.5 h. The intervention mixtures listed in the table were placed in the right lower lung (RLL) through an epidural catheter placed down the working channel of a bronchoscope (Vision Sciences BRS-5000, Laborie, US) at time = 0. The OP (dimethoate EC40) intervention mixture contained 1/25^th^ a severe poisoning dose used in previous studies [15]. The left lung was isolated during pulmonary instillation through use of a bronchial blocker. Bronchoscopy, bronchoalveolar lavage (BAL) and pulmonary biopsy for both direct (right) and indirect (left) lungs took place at 24.5 h and 48 h, after which the minipig was euthanized. (A) Breathing hoses from Siemens Servo 300A ventilator; (B) bronchoscope dual-axis swivel adapter; (C) bronchial blocker inflation line; (D) endotracheal tube cuff inflater; (E) torque controlled bronchial blocker (TCB; Univent endotracheal tube); (f) right accessory bronchus; (g) bronchial blocker cuff; (h) bronchoscope (BRS-5000). Diagram illustrating the instrumentation used is reproduced from previous work [18]. edc: epidural catheter.

Animals (groups *n* = 5) were randomized to sham bronchoscopy (sham control; bronchoscopy with *no* pulmonary instillation of mixtures) or to receive 0.5 mL/kg of: 0.9% NaCl (saline control), porcine gastric juice (GJ), GJ with OP insecticide (dimethoate 40% emulsifiable concentrate [EC40]; Cheminova, Denmark) (OP + GJ), or GJ with solvent (cyclohexanone (Sigma-Aldrich, UK)) (Solv + GJ) into the isolated right lung under bronchoscopic guidance at the beginning of the experiment (Figure 1). Intravenous oleic acid (OA; Sigma-Aldrich, UK) (0.08 g/kg) was administered to one animal as a positive control for assays. Fresh porcine gastric juice was obtained from an abattoir, large particulates removed with gauze, pH titrated to 2.0 [19] with HCl, microbes treated with antibiotic and antifungal treatment and stored at −80 °C (Figure S4). Animals were randomized within their groups to either a computed tomography (CT) scan (*n* = 12) or no scan (*n* = 14). One minipig in the saline group developed a pneumothorax before 48 h; some 24–48 h data from this animal were omitted before thoracocentesis and subsequent euthanasia at 48 h.

### Bronchoalveolar lavage fluid, blood and plasma

Figure 1 shows sampling timepoints for blood, preparation of plasma, bronchoalveolar lavage fluid (BALF), bronchial lung biopsy (data not shown) and pulmonary CT. Mean (±SD) BALF return volume was 56 ± 16% (*n* = 26) with cellular viability 85 ± 11% (*n* = 20). BALF was processed for cells (number/type) [20], protein (Bradford assay) [21], albumin (Olympus Diagnostics Ltd, Watford) and surfactant protein-D content (Neobiolab, MA). Cytokines (IL-6, IL-8) and CRP were measured by ELISA (R&D systems, UK). Separate tracheal secretion samples were taken at −30 min and BALF samples at 48 h for microbiology. Arterial blood was analysed for PO_2_, PCO_2_, lactate, glucose and electrolytes (EPOC, Woodley Equipment, UK) and venous blood for AChE activity [22].

### Computed tomography (CT)

CT scans (16 slice GE Lightspeed, Buckinghamshire, UK) were performed at regular intervals (Figure 1) in dorsal recumbency during paused ventilation with PEEP 5 cmH_2_O. Hand-drawn outlines of 2D lung slices were propagated into a 3D image with voxel data analysed (Analyze 10.0, US) for density spread. Percentage of poorly/non-aerated lung tissue ([voxels between −499 and +250 [23–25] Hounsfield Units (HU)/voxel number between −999 and +250 HU] × 100) within the 3D image was calculated for each lung at each timepoint.

### Histopathology

Sampling of cranial and caudal regions of both right (directly injured) and left (indirectly injured) lungs was performed post-mortem (see online supplement) for each animal to assess the severity of regional lung injury [26].

### Statistics

The study was powered to show histopathological differences between aspiration lung injury and controls, not with other treatment groups, using previously obtained porcine pulmonary histopathological data [26]. Actual study histopathology scores from right sham versus OP + GJ lungs at 48 h (*n* = 5, 5.3 ± 3.6 versus 11.7 ± 4.6 points, respectively) showed 80% power with an alpha error of 10%.

Omnibus testing was performed using either Friedman or Kruskal-Wallis tests. If these were significant, further post hoc analysis using Dunn’s post-test or permutation testing [27] was performed (see online supplement for details).

## Results

### Porcine aspiration model of acute lung injury

**Figure 2.**
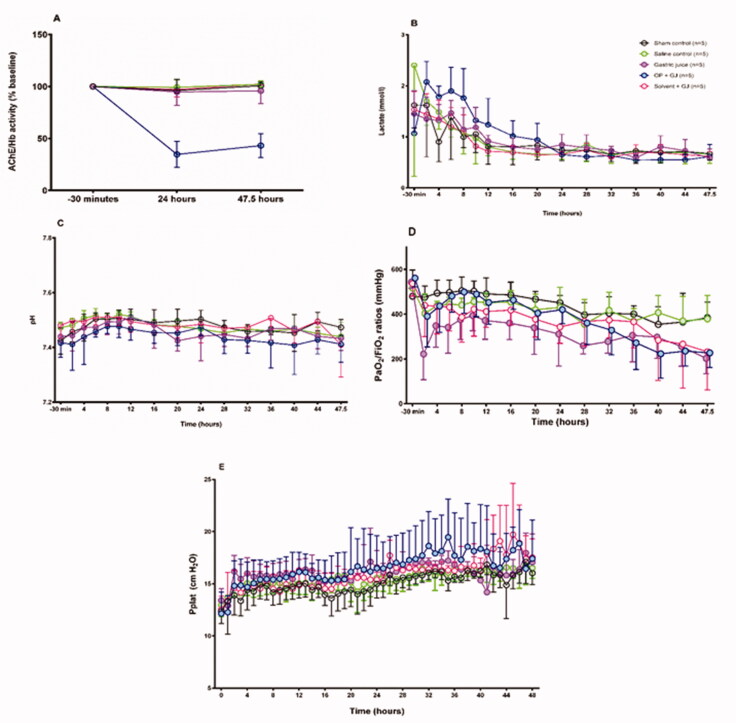
Effects of pulmonary treatments on red cell AChE, cardiovascular function and lung function. (A) red cell AChE activity (relative to Hb concentration, normalized to baseline) showing effect of OP insecticide in the OP + GJ group; (B) arterial plasma lactate concentration (mmol/L) and (C) arterial pH showing no cardiovascular effects of the instilled mixtures; (D) PaO_2_/F_i_O_2_ ratios and (E) plateau airway pressures (Pplat). By 48 h, the intervention groups had developed a mild-moderate acute respiratory distress syndrome (ARDS) with PaO_2_/F_i_O_2_ ratios of 227 (±65) mmHg, 203 (±69) mmHg and 232 (±171) mmHg for OP + GJ (n = 4), G (n = 5) and Solv + GJ-treated pigs (n = 5) respectively (compared to 385 (±70) mmHg (sham bronchoscopy, n = 5), 317 (±166) mmHg (saline controls, n = 5) NB. The PaO_2_, pH and lactate data for one OP+GJ pig at 47.5 h was missing likely due to equipment failure. For presentation, Figures 2D and 2E omit data between 24–48 h from one saline pig that developed a pneumothorax. Please see online methods supplement for more information on data management. The graphs show mean with SD.

Reduced AChE activity was only found in the OP + GJ group (Figure 2(A)) confirming OP poisoning. All but sham pigs showed early acute lung injury (Figure 2(D)), whilst remaining were haemodynamically stable (Table 1, Figure 2(B,C)). PaO_2_/F_i_O_2_ ratios were significantly reduced in pigs receiving OP + GJ (*p* < 0.05), Solv + GJ (*p* < 0.01), or GJ (*p* < 0.0001) versus sham controls over 48 h (Figure 2(D)). Plateau pressure (Pplat) increased over time (Figure 2(E)).

**Table 1.** Cardiovascular physiological data.

Group	MAP (mmHg)	CO (L/min)	SVR (Dyne/s/cm^5^)
**Sham**	75 (4.4)	3.2 (0.7)	2019 (565)
**Saline**	84 (6.1)	2.9 (0.4)	2393 (447)
**GJ**	77 (4.7)	3.4 (0.8)	2007 (478)
**Solv + GJ**	76 (5.8)	3.6 (1.0)	2006 (380)
**OP + GJ**	70 (4.9)	3.7 (1.0)	1589 (307)

Values show mean (SD) for the duration of the experiment (0 to 48 h). Groups had significant differences (*p* < 0.0001) when using Friedmann analysis for MAP, CO and SVR. The OP + GJ group had the lowest mean arterial pressure (MAP) and systemic vascular resistance (SVR) secondary to OP-induced vasodilatation. Intravenous norepinephrine was only used in two pigs to increase MAP (one OP + GJ pig at a low dose (80–800 µg/h) for first 2 h, and one positive control pig receiving IV oleic acid; data not shown in graphs/tables).

MAP: mean arterial pressure; SVR: systemic vascular resistance; CO: cardiac output.

### Bronchoalveolar lavage fluid analysis

**Figure 3.**
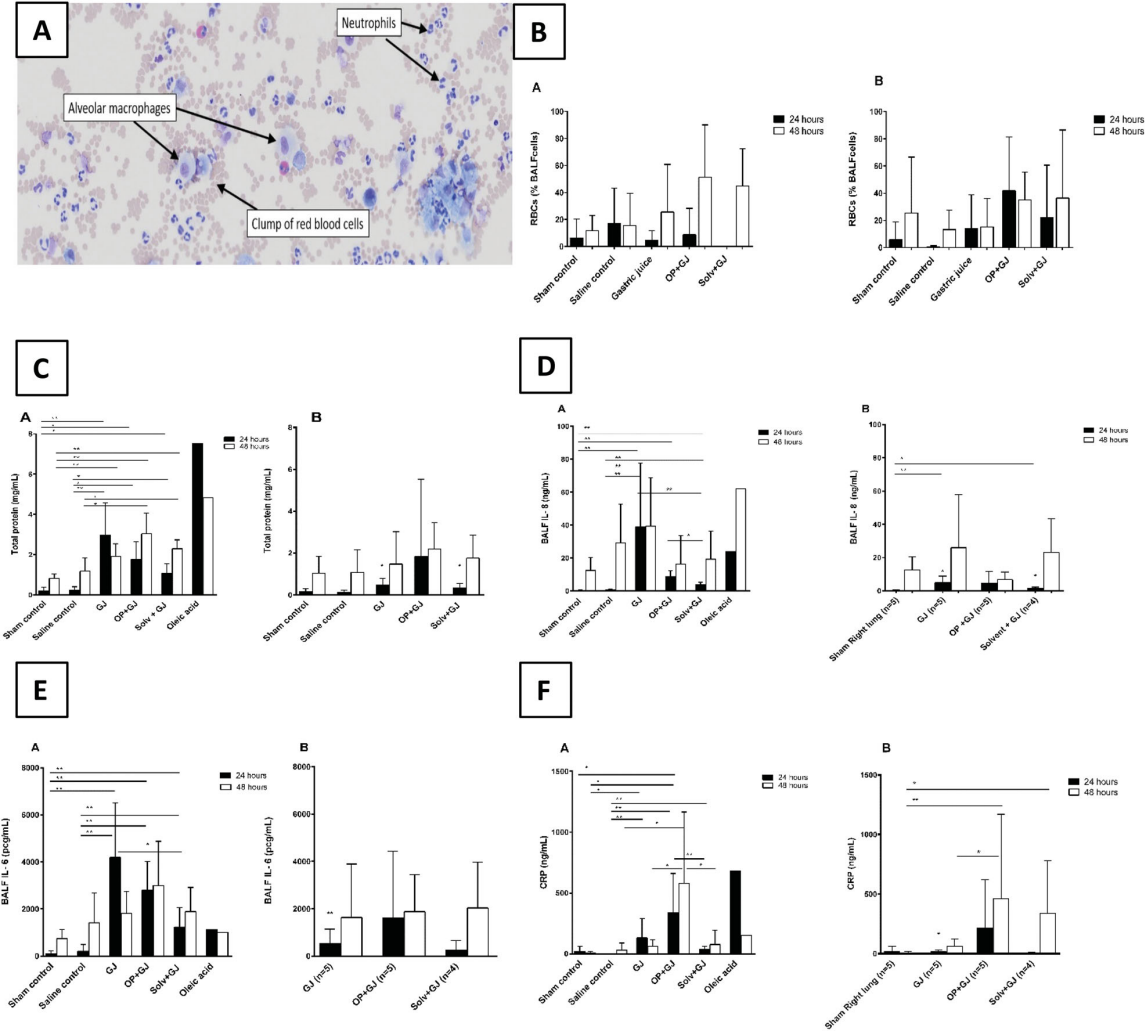
Effects of pulmonary treatments on BALF and plasma markers of inflammation. (A): BALF cellular contents from a representative OP + GJ directly-injured (right) lung at 48 h. This cytospin image shows the presence of numerous neutrophils with the occasional alveolar macrophage. Many red blood cells can also be seen. Cells have been stained with Diffquik® (image taken using Olympus slide scanner VS120 at 20×). (B) The presence of red blood cells in the BALF from minipigs receiving pulmonary treatments. Percentage of red blood cells (RBC) in a count of 100 cells within the BALF of samples at 24 and 48 h in the (B–A) directly-injured and (B-B) indirectly-injured lungs. No statistical significance was found between groups. (C) Protein concentrations in the BALF of minipigs receiving pulmonary treatments. BALF total protein in (C–A) directly-injured and (C–B) indirectly-injured lungs at 24 and 48 h. There was a significant difference between directly-injured lung groups at both time points, but not indirectly-injured lungs. The asterisks indicate that there was significant difference between directly- and indirectly-injured lungs of GJ and Solv + GJ pigs at 24 h. (D) BALF interleukin 8 (IL-8) concentrations in (D–A) directly and (D–B) indirectly-injured lungs at 24 and 48 h. There was a significant difference for both directly- and indirectly-injured lung groups at 24 h but not 48 h. Comparisons between directly- and indirectly-injured lungs found that IL-8 was significantly lower in the indirectly-injured lungs of GJ and solv + GJ pigs (asterisks no bar). (E) BALF interleukin 6 (IL-6) concentrations in (E–A) directly-injured and (E–B) indirectly-injured lungs at 24 and 48 h. Only directly-injured lungs at 24 h were significantly different between groups. Comparisons between directly- and indirectly-injured lungs found that IL-6 was significantly lower in the indirectly-injured lung of only GJ pigs at 24 h (asterisk no bar). (F) BALF C-reactive protein (CRP) concentrations in (F-A) directly-injured and (F-B) indirectly-injured lungs at 24 and 48 h. There was a significant difference between directly-injured lung groups at 24 h, and directly and indirectly-injured lungs at 48 h. Comparisons between directly and indirectly-injured lungs found that CRP were significantly lower in the indirectly-injured lung of only GJ pigs at 24 h (asterisk no bar). (B–F) show mean and SD. When there were significant differences between sham control and a treatment group in the indirectly-injured lungs, these were illustrated on the graph (Figure 3D-B Right panel; 3F–B Right panel).

Alveolar lavage demonstrated neutrophilic inflammation and haemorrhage in the right (directly-injured) lungs following instillation of OP + GJ (Figure 3(A)). There was no significant difference in blood neutrophil numbers between groups (results not shown). The highest mean right-lung RBC count was in OP + GJ pigs at 48 h (51%±39% of 100 cells counted) with no statistical differences between groups due to marked variation in the small sample (Figure 3(B), Table S1). OP + GJ, GJ and Solv + GJ treated pigs had a greater percentage of white blood cells (WBCs) that were neutrophils in the right lung at 24 h than sham control pigs (*p* < 0.01) (Figure S2, Table S1), but this difference had disappeared by 48 h. In left (indirectly-injured) lungs, OP + GJ had the greatest percentage of WBCs that were neutrophils at 24 and 48 h. GJ and Solv + GJ, but not OP + GJ, treated pigs had lower percentages of neutrophils in the left versus right lungs at 24 h (*p* < 0.05). No significant change was seen in blood neutrophil count (data not shown).

Protein and albumin concentrations were statistically greater in BALF of animals following instillation of GJ mixtures at both 24 and 48 h for the right, but not left, lungs versus sham controls (Figure 3(C), Figure S3). At 24 h, GJ-treated pigs had the highest protein concentrations while, at 48 h, OP + GJ-treated pigs had the highest protein concentrations in the right lungs (Table S1). Both lungs of all animals had markedly higher protein concentrations at 48 h possibly showing the effects of BAL, bronchial biopsy and artificial ventilation. Alveolar protein was non-significantly lower in Solv + GJ-treated pigs than OP + GJ and GJ pigs at 24 h and OP + GJ at 48 h. No change in BALF pulmonary surfactant protein D was noted across the groups (Table S1).

Inflammatory cytokines were markedly raised in the lungs of pigs given GJ mixtures but not in their plasma (data not shown), indicating localized pulmonary inflammation. At 24 h, BALF IL-8 and IL-6 concentrations were raised in the right lung of OP + GJ, GJ and Solv + GJ-treated groups versus sham/saline controls (Figure 3(D,E); Table S1), in particular in those receiving GJ alone (mean IL-8 concentrations at 24 h (39 ± 39ng/mL) and 48 h (39 ± 30ng/mL)). Solv + GJ-treated pigs had the lowest BALF IL-8 concentrations in directly-injured lungs (4 ± 2 ng/mL) at 24 h.

The highest mean BALF IL-6 concentration was found in the rights lungs of GJ-treated pigs at 24 h (4200 pcg/mL) and OP + GJ pigs at 48 h (3000 pcg/mL). Solv + GJ pigs had significantly less (*p* < 0.05) BALF IL-6 in the right lung when compared with GJ pigs at 24 h.

At 24 h, CRP was raised in the right lungs of pigs given all GJ mixtures (*p* < 0.01) when compared with saline controls (Figure 3(F)), but raised only in the plasma of GJ-treated pigs (data not shown). It was markedly higher in lungs directly-injured by OP + GJ at 48 h. Solv + GJ pigs had significantly less BALF CRP versus OP + GJ treated pigs at 24 h (*p* < 0.01) and 48 h (*p* < 0.05) in directly-injured lungs. In the left, indirectly-injured lungs, BALF CRP was substantially higher at 48 h in OP + GJ (*p* < 0.01) versus sham control (right lung) or GJ pigs (*p* < 0.05).

### BALF microbiology

BALF samples taken at 48 h were cultured for aerobic bacteria. Predominant species found were *Escherichia coli* (35%), DNAase-negative *Staphylococcus* spp. (22%) and *Klebsiella pneumoniae* (*17*%). The BALF bacteria count was >10^4^ cfu/mL at 48 h in sham control (*n* = 2/5), saline control (*n* = 3/5), GJ (*n* = 3/5), OP + GJ (*n* = 3/5) and Solv + GJ (*n* = 3/5) pigs (Figure S5). There was no significance when comparing BALF samples taken at 48 h across all groups.

### Lung injury seen on CT imaging

**Figure 4.**
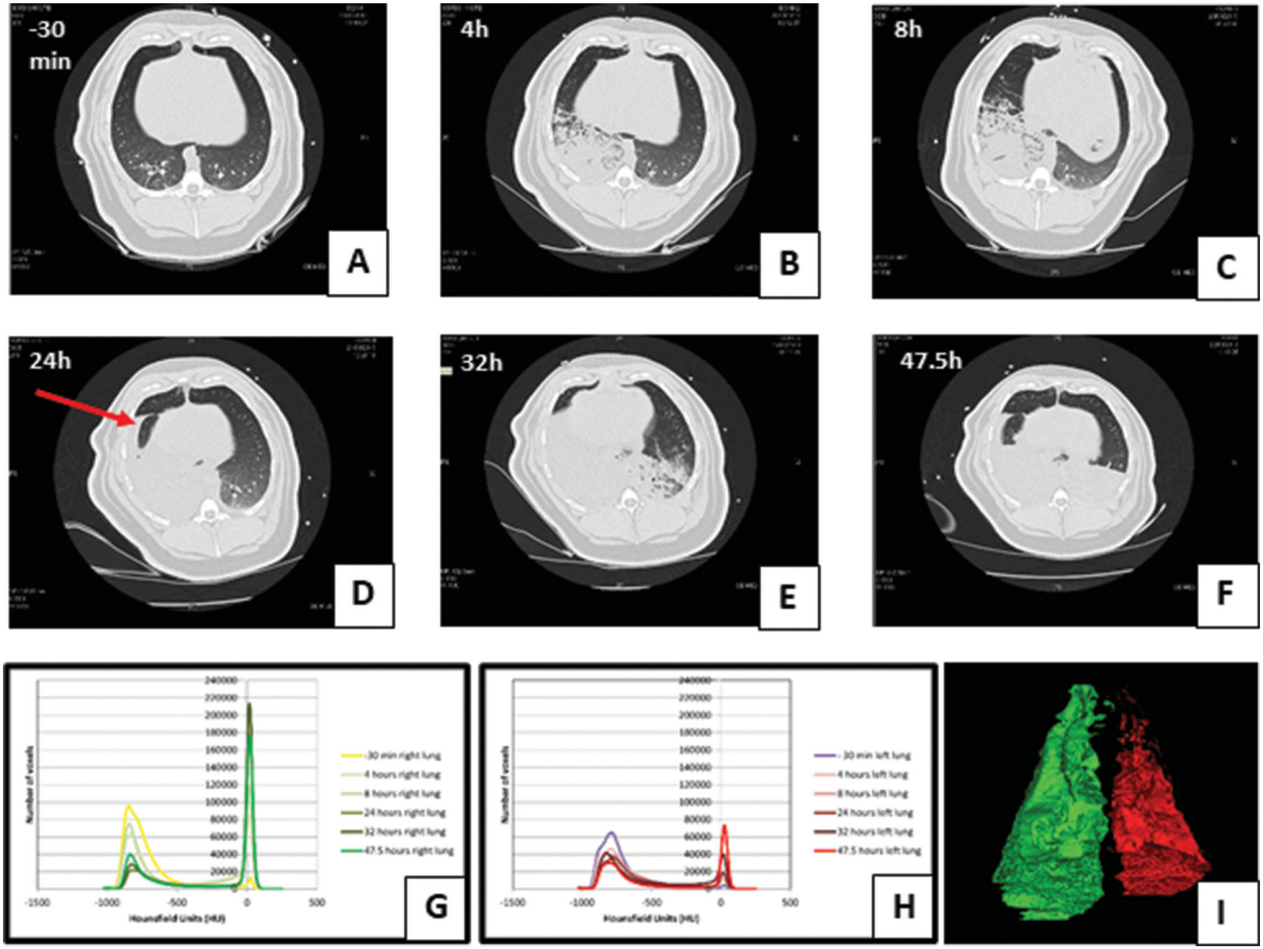
Lung injury caused by instillation of OP + GJ shown through serial CT scans and lung density analysis. Serial CT scans of an OP + GJ pig before (A) and after (B–F) instillation of a mixture of OP + GJ into the right lung of a minipig at time = 0, with voxel density analysis of the directly-injured (G) and indirectly-injured (H) lungs over time. Panel I shows only the non-aerated voxels (–250 to +250 HU) 47.5 h after instillation of OP + GJ in the right, directly-injured lung compared with the left, indirectly-injured lung. By 4 h (B), there is an obvious right-sided lower area of consolidation which enlarges over time to involve most of the right lung by 24 h (D) with a large pleural effusion seen in the fissure (arrow). By 32 h, the contralateral left lung is also involved showing dorsal consolidation (E) which worsens with bilateral pleural effusions at 47.5 h (F). The right lung recovers slightly at this time with more aeration sub-sternally (anteriorly). Indirectly-injured (left) lung (H) is represented in red, directly-injured (right) lung (G) in green in the online version. Directly-injured lung has a peak amount of poorly aerated lung at 32 h, which then reduces a little by 47.5 h, mirroring the CT images. The indirectly-injured lung has less volume than the contralateral lung at baseline, hence the smaller peaks. However, the spread of voxels shows a good proportion of well-ventilated, aerated lung at 47.5 h, unlike in the directly-injured lung.

**Figure 5.**
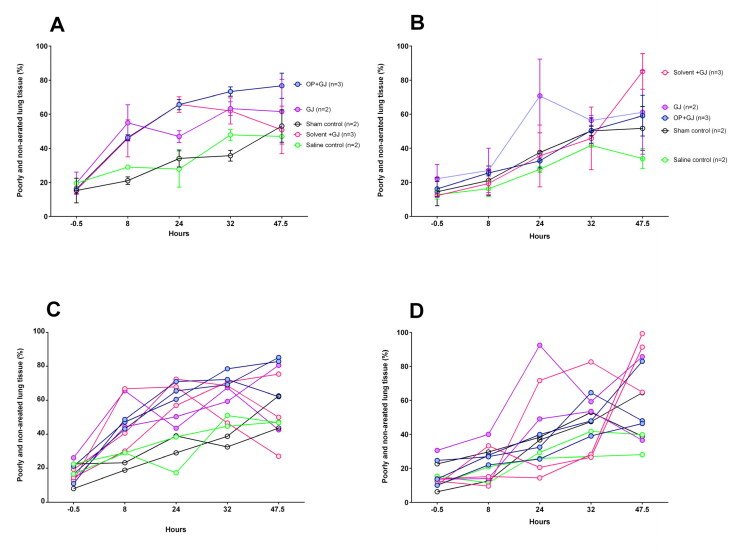
Change in proportion of poorly/non-aerated lung tissue over time by groups. Graphs showing the mean ± SEM percentage of poorly/non-aerated lung tissue (–499 to +250 HU) from (A) directly-injured right and (B) indirectly-injured left lungs over 48 h. Individual lung data are shown in dot plots for the (C) directly-injured right and (D) indirectly-injured left lungs over 48 h. Data from one saline control pig left lung at 32 h has been omitted because of a large pneumothorax which was drained before the pigs 47.5 h CT scan.

CT analysis allowed the lung injury caused by pulmonary instillation of treatments to be measured in representative animals (*n* = 12) over 48 h (Figure 4). Pulmonary instillation of both GJ and OP + GJ caused a notable increase in the percentage of poorly/non-aerated right lung tissue at 24 and 47.5 h when compared with sham controls (Figure 5(A), Table 2). OP + GJ-treated pigs had the greatest proportion of such dense right lung tissue at 47.5 h (77 ± 13%), with saline controls having least (47 ± 0.2%). By 24 h, Solv + GJ, OP + GJ and GJ treatments had similar effects, but aeration improved more rapidly in pigs exposed to pulmonary Solv + GJ. By 47.5 h, the Solv + GJ-treated pigs had markedly less poorly/non-aerated lung tissue (51 ± 24%) than either GJ (62 ± 27%) or OP + GJ (77 ± 13%) pigs and were similar to sham or saline controls (53 ± 13%, 47 ± 0.2%, respectively).

**Table 2.** Percentage (mean ± SD) of poorly and non-aerated lung tissue in the directly-injured lung at time –30 min, 24 and 47.5 h.

Groups	−30 min	24 h	47.5 h
Sham (*n* = 2)	15.3 (10.2)	34 (7.1)	53.1 (13.4)
Saline (*n* = 2)	19.6 (4.2)	27.8 (14.9)	47 (0.2)
GJ (*n* = 2)	19.6 (9.3)	46.9 (4.8)	61.6 (26.9)
OP + GJ (*n* = 3)	16.2 (5.2)	65.7 (5.2)	76.7 (12.7)
Solv + GJ (*n* = 3)	15.5 (3.4)	65.7 (7.9)	50.8 (24.2)

The left lungs also slowly increased in density over time with less clear differences between groups (Figure 5(B)).

### Lung histology and electron microscopy

**Figure 6.**
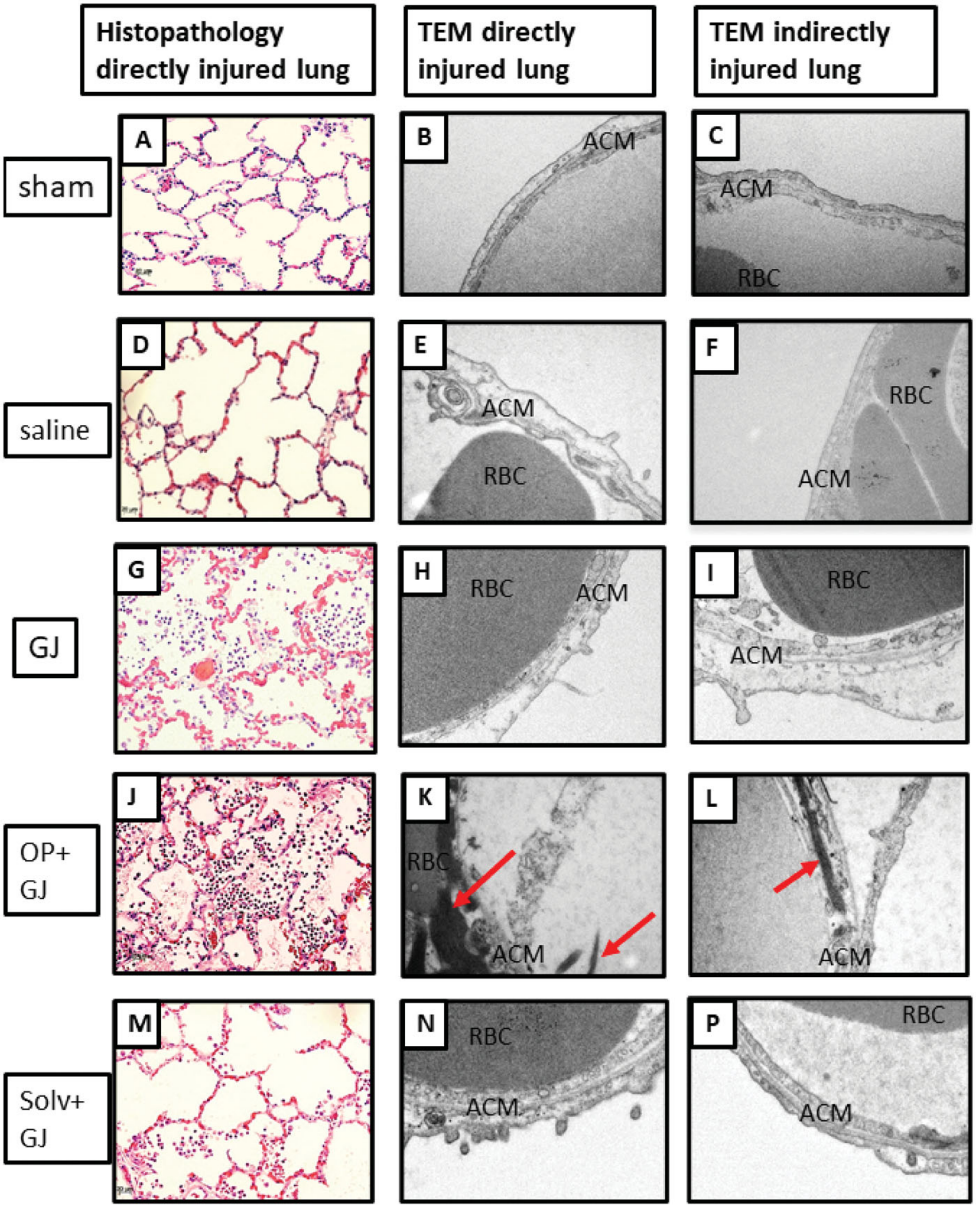
Effects of pulmonary instillation of mixtures of GJ, OP + GJ and Solv + GJ on minipig lung. Comparison of histopathological pig lung architecture in representative sections 48 h after sham bronchoscopy (A/B), administration of saline (D/E), GJ (G/H), OP + GJ (J/K) or Solv + GJ (M/N) into directly-injured right lower lungs. Direct injury with GJ and with OP + GJ caused alveolar and interstitial oedema, neutrophil infiltration, haemorrhage, fibrin deposition, vascular congestion, and necrosis. Haemorrhage and necrosis were more pronounced in the OP + GJ lungs while Solv + GJ caused less lung injury. On electron microscopy, direct injury with GJ, OP + GJ and Solv + GJ shows some alveolar capillary membrane swelling; the alveolar membrane component peels off into the alveolar space in GJ and OP + GJ groups. OP + GJ also led to fibrin deposition (arrows) in and around the alveolar capillary membrane. In lungs indirectly-injured by GJ (I) and OP + GJ (L), alveolar injury can be seen. Images edited in PowerPoint (Microsoft 2020). Original magnification: 20× for light microscopy, 25,000× for electron microscopy. ACM: alveolar capillary membrane; RBC: red blood cell.

**Figure 7.**
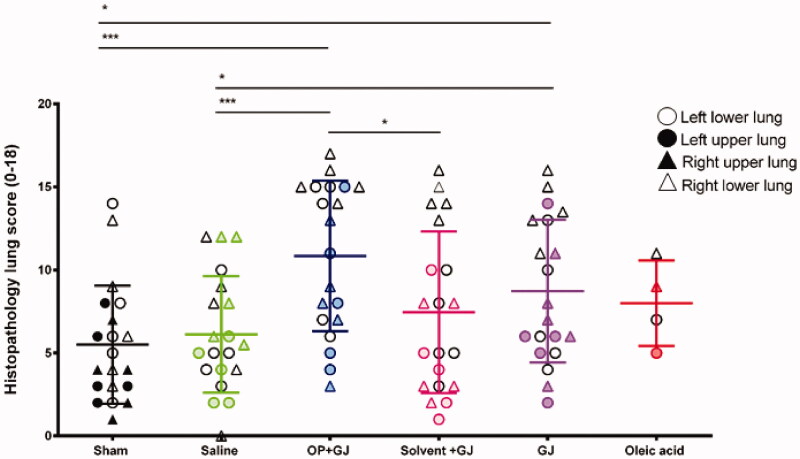
Dot plot showing the spread of histopathology scores in cranial and caudal (upper and lower) parts of the lung in all groups. Triangles indicate directly-injured right lungs, circles indicate indirectly-injured left lungs. Empty triangles or circles denote lower, as opposed to upper, lung samples. Most severe injury was noted in the directly-injured right caudal (lower) lung samples (empty triangles) in the intervention groups. Mean and SD shown.

Pulmonary OP + GJ instillation created significant airway and interstitial neutrophil infiltration, haemorrhage, oedema and necrosis with fibrin deposition (Figure 6(J–L)). The four lung replicate samples (upper/lower, right/left lung) from each animal described the distribution of lung injury (Figure 7). Using each replicate as a single data point, analysis of both lungs together showed that GJ (*p* < 0.05) and OP + GJ (*p* < 0.01)-treated animals had significantly more damage when compared with sham or saline controls (Table S2). There was no statistical difference between GJ and OP + GJ for lungs combined or individually. Scoring for both solv + GJ lungs combined was significantly lower than both OP + GJ-treated lungs (*p* < 0.05). Solv + GJ left lung histopathology scores were also lower than OP + GJ-treated left lungs (*p* < 0.05), as seen on microscopy (Figure 6 (L/P)).

## Discussion

Pulmonary instillation of OP + GJ created a more severe lung injury when compared with sham controls or Solv + GJ-exposed lungs. Although the study was not powered to reveal differences between active aspiration groups, pulmonary instillation of OP + GJ created subtly different, and in some cases more severe direct and indirect lung injuries when compared with GJ aspiration.

For this study, we established a large animal model of OP insecticide poisoning and aspiration [18] in which the poison was carefully administered into only one lung, without any systemic exposure except that caused by absorption from within the lung. To begin to understand the role of individual pesticide components being aspirated, we examined the effects of a formulated agricultural OP insecticide combined with gastric contents, gastric contents alone, and gastric contents with the main solvent from the insecticide, versus a sham bronchoscopy or instillation of saline placebo. We found that the dose of OP insecticide (with GJ) instilled into a lung caused modest inhibition of red cell AChE but a severe lung injury characterised by haemorrhage, neutrophil inflammation and pro-inflammatory cytokines, with worsening oxygenation over 48 h but no cardiovascular effects.

The severity of lung injury noted might partly explain the high case fatality seen in patients who require tracheal intubation on arrival at hospital and who have commonly aspirated their stomach contents, including the insecticide.

We also found injury in the lung that was not directly damaged. Some of this indirect injury may have been due to mechanical ventilation (despite the use of lung-sparing strategies) and repeated bronchoscopy (lavage/biopsy) [28] over 48 h. However, indirect injury was more severe in animals receiving OP insecticide and GJ, suggesting a systemic effect from the pesticide or the inflammatory response to lung injury. Compared to the lung changes, systemic inflammation (neutrophilia, cytokines and CRP) was not prominent. The increased severity in lungs receiving insecticide versus solvent suggests that either the insecticide or surfactants in the formulation are responsible.

Analysis of the contents of the alveolar fluid revealed that GJ alone caused the most severe injury at 24 h in terms of cell number, concentrations of protein and cytokines (IL-6/8). However, by 48 h, the injury had markedly worsened in lungs directly injured with OP + GJ, suggesting an ongoing inflammatory process.

Gastric acid aspiration inflicts a two-hit injury, consisting of an immediate chemical acid burn that occurs seconds to hours after aspiration, followed by inflammation peaking at 4–6 h [16,29] often causing high concentrations of lung IL-8 [19,30]. Therefore, samples of BALF at 24 h and 48 h may have missed an earlier peak of pulmonary inflammation. However, from the BALF data that we obtained, the injury in GJ-treated lungs endured past 4–6 h, peaking at 24 h, indicating injury from not only acid but also probably small particulates. In the pigs receiving OP + GJ, the injury was still developing at 48 h, suggesting another cause of injury in addition to the acid and particulate lung injury, e.g., the toxic effect of OP and/or its formulation contents (surfactant or solvent) or simply secondary infection. The relatively less severe injury in pigs receiving Solv + GJ suggests that solvent was not the cause of this additional injury.

Lung CT analysis showed ongoing injury after 24 h in pigs receiving OP + GJ, with the highest mean percentage of poorly/non-aerated lung tissue of any group noted in their right lungs at 32 h and 47.5 h. At the latter time, pulmonary histopathology revealed worse damage in direct OP + GJ injured lungs than GJ and Solv + GJ pig lungs. Lack of a significant difference between GJ and OP + GJ right lungs at 47.5 h may be due to inadequate study power, short study duration and/or the model itself.

OP insecticide and gastric contents also resulted in greater pulmonary haemorrhage in directly-injured lungs at 48 h than other injuries, the presence of blood being confirmed by histopathology and BALF RBC counts. Previous studies have found that orogastric administration of OP insecticide causes pulmonary oedema and haemorrhage in animals [26,31] and humans [32], likely due to increased acetylcholine-dependant endothelial permeability [33] and alveolar destruction [31]. Inhaled OP also produces alveolar thickening and destruction with increased capillary congestion and extravasated RBCs in rats [34]. Blood probably resulted from extravasation damage to alveoli (breaks in alveolar capillary membrane), parenchyma or bronchi secondary to a toxic or direct chemical effect. Modest haemorrhage was also noted in control animals after bronchoscopy and biopsy, so presence of blood in animals treated with OP + GJ may not be entirely attributable to these treatments.

Several BALF samples at 48 h had a bacterial count >10^4^ cfu/mL. Pigs are prone to respiratory disease and pneumonias [35]. These Gottingen minipigs were reared in a barrier facility that carefully monitors animals for infection. However, the tracheal and BALF samples did not grow bacteria known to be associated with porcine respiratory infections [35] but *E. coli* and *K. pneumoniae,* well-known sources of human VAP [36]. It is unknown whether these bacteria grew because of contamination from the (ex-human ICU) ventilator, respiratory equipment, or the research team themselves, although measures were taken to reduce these possibilities.

Humans exposed to occupational OPs are more prone to respiratory tract infections [37], so it may be that patients who ingest and aspirate OP + GJ are more prone to aspiration pneumonia than normal ICU patients, explaining in part the increased mortality in intubated OP-poisoned patients secondary to OP self-poisoning. The role of the cholinergic anti-inflammatory pathway [38] in suppressing inflammation due to OP insecticide-induced cholinergic overstimulation requires further investigation [39].

The study raises the question of whether the cyclohexanone solvent could be protective since Solv + GJ placed in the lungs unexpectedly caused less damage than GJ or OP + GJ. Material safety data sheets state that inhaled cyclohexanone can cause respiratory tract oedema and chemical pneumonitis; cyclohexanone is also synergistically toxic in the context of dimethoate EC40 ingestion [15]. Our findings suggest that cyclohexanone may exert an anti-inflammatory and antibacterial [40] effect on the alveolar epithelium.

### Limitations

We acknowledge that there are many different types and forms of OP insecticides, with unique concentrations of chemicals to aid dispersal (e.g., surfactants, solvents, anti-foaming agents and unspecified chemicals) which limit the interpretation of our results. This study also did not address the co-ingestion of other potentially harmful substances often consumed during a human self-poisoning episode, e.g., alcohol [41] and/or other toxins/insecticides. Instead, this study created aspirates deliberately standardized for one type of organophosphorus insecticide (dimethoate EC40), pH, microorganisms, particulate matter and volume to understand the pathophysiology from this unique aspiration lung injury under standard mechanistic conditions between groups.

The experimental model was designed to give groups the same total aspiration volume, not the same volume of GJ. However, the difference in volume for the GJ groups was only a few mL i.e., 30 kg pig: OP + GJ 12 mL, Solv + GJ 13.7 mL versus 15 mL of gastric juice in the GJ group. There were other GJ factors that were not standardized, e.g., enzyme content, which may have affected outcomes. Also, despite being intubated, the pigs did not have their own stomachs emptied and could be a confounding factor.

The pharmacodynamics concerning the activation of dimethoate EC40 into the oxon compound (causing AChE inhibition) in the lungs using this model is beyond the scope of this paper. However, further work on this may elicit clues as to the apparent ongoing pulmonary toxicity in the OP + GJ group at 48 h.

This study created a unique patterned lung injury due to the model design and deliberate placement of treatments in the right lower lung. In order to appreciate these differences, the histopathology sample replicates taken from bilateral cranial and caudal lungs were treated as individual data points. While this practice can lead to a *pseudoreplication* [42] error due to an assumed sample variance secondary to random factors, we felt that this practice was warranted in order to make use of this expensive and valuable animal model.

Overall, the data and experience collected during this study should allow future larger, and longer, animal studies to be designed allowing observation of longer term lung injuries.

## Conclusion

Pulmonary instillation of the organophosphorus insecticide, dimethoate EC40, and gastric juice created more lung damage than controls or the solvent, cyclohexanone and gastric juice. Our findings have relevance to human poisoning where pre-hospital aspiration in patients with reduced consciousness is common [5,43], increasing the duration of hospital stay, morbidity, case fatality [44] and cost to health services. The evidence here supports early airway protection with intubation [45] in OP insecticide self-poisoned patients with reduced consciousness and in those receiving gastric lavage to prevent a concomitant direct and indirect aspiration lung injury.

## Supplementary Material

Supplemental Material
