# Supplementary material for: Lung injury caused by aspiration of organophosphorus insecticide and gastric contents in pigs
Source: Clin Toxicol (Phila). 2022 Feb 11;60(6):725–36. doi: 10.1080/15563650.2022.2028803 (PMC9162497; doi:10.1080/15563650.2022.2028803)
Supplement: Supplemental Material [file ICTX_A_2028803_SM7780.docx]

**Title:** **Lung injury caused by aspiration of organophosphorus insecticide and gastric contents in pigs.**

**Online Data Supplement**

**Supplementary results**

**Tables**

**Table S1**

| Groups | RBCs (% of 100 BALF cells) | Cell numbers x 10^7 NB^ | Neutrophils (% of BALF WCs) | Total protein (mg/L) | Albumin (mg/L) | SP-D  (ng/mL) | IL-6  (pcg/mL) | IL-8  (ng/mL) | CRP  (ng/mL) |
| --- | --- | --- | --- | --- | --- | --- | --- | --- | --- |
| Sham 24 hrs (D) | 6.3 (14) | 3.6 (4.7) | 13.8 (10.9) | 195.8 (182.8) | 53.6 (64.8) | 177 (54.3) | 101(132.8) | 0.34 (0.31) | 20.1 (42.1) |
| Sham 24 hrs (I) | 5.8 (13) | 5.3 (3.9) | 4.0 (4.4) | 156 (142.4) | 61.4 (62.8) | n/a | n/a | n/a | n/a |
| Sham 48 hrs (D) | 11.8 (11.2) | 12 (8.8) | 63 (15.0) | 813 (232.4) | 187.2 (81.7) | 68.6 (15.3) | 743.7(377.1) | 12.52 (7.8) | 7.5 (10.1) |
| Sham 48 hrs (I) | 25.4 (41.3) | 15 (6.9) | 56.3 (27.3) | 1031 (818.4) | 208.8 (209.4) | n/a | n/a | n/a | n/a |
| Saline 24 hrs (D) | 17.1 (26) | 3.6 (2.9) | 20.1 (22.4) | 226.6 (176.4) | 85.4 (70.1) | 111.1 (22.2) | 206.1 (284.2) | 0.57 (0.6) | 1.6 (1.0) |
| Saline 24 hrs (I) | 0.4 (0.9) | 3.1 (2.5) | 19.2 (12.9) | 137.0 (101) | 60 (49.3) | n/a | n/a | n/a | n/a |
| Saline 48 hrs (D) | 15.5 (23.8) | 18.3 (10) | 55.3 (25.8) | 1178 (671.8) | 311 (202.8) | 70 (29.3) | 1419(1255) | 29.04 (23.9) | 29.8 (59) |
| Saline 48 hrs (I) | 13.2 (14.2) | 14.2 (11.9) | 43.3 (24.9) | 1076 (1080) | 218 (219.3) | n/a | n/a | n/a | n/a |
| GJ 24 hrs (D) | 4.7 (7) | 16.2 (9.4)*, # | 71.2 (8.9)**, ## | 2967 (1619)**, ## | 734.4 (368.1)*, # | 79.9 (56.2) | 4189 (2323)**, ## | 38.8 (38.9)**, ## | 130.1 (158)## |
| GJ 24 hrs (I) | 14.1 (24.6) | 20.6 (36.2) | 27.9 (19.9) | 464 (334.2) | 153.6 (130) | n/a | 531.9 (603.4) | 5.0 (3.9)** | 16.7 (16.0) |
| GJ 48 hrs (D) | 25.5 (35.4) | 33.5 (21.8) | 70.3 (10.0) | 1916 (630.5)** | 555.2 (237.7)* | 51.3 (13.5) | 1820 (923.7) | 39.2 (29.7) | 61 (53.1)* |
| GJ 48 hrs (I) | 15.2 (21) | 14.3 (9.7) | 65.3 (20.2) | 1475 (1544) | 280.8 (328.4) | n/a | 1629 (2255) | 26.0 (31.8) | 62.3 (59.5) |
| OP+GJ 24 hrs (D) | 8.7 (19.5) | 10.2 (7.0) | 72.7 (11.2)**, ## | 1751 (902.3)*, # | 549.6 (297.8)*, # | 102.5 (21.6) | 2796 (1228)**, ## | 8.9 (3.4)**, ## | 337.2 (323.6)*, ## |
| OP+GJ 24 hrs (I) | 41.8 (39.5) | 7.23 (4.2) | 39 (32.8) | 1842 (3692) | 355.2 (625.6) | n/a | 1615 (2822) | 4.6 (7.0) | 215.7 (404.8) |
| OP+GJ 48 hrs (D) | 51.2 (38.9) | 46.3 (21.7) | 69.0 (17.0) | 3032 (1036)**, # | 776.8 (211.8)**, # | 48.7 (13.7) | 2993 (1884) | 16.2 (17.4) | 580.9 (584.3)*, #, $ |
| OP+GJ 48 hrs (I) | 35 (20.4) | 24.3 (13.1) | 84.3 (3.7) | 2194 (1259) | 499.2 (236.7) | n/a | 1877 (1573) | 6.7 (4.7) | 462.9 (706.9)**, $ |
| Solv+GJ 24 hrs (D) | 0 (0) | 14.4 (11.4)# | 68.1 (17.1)**, # | 1059 (494.7)*. # | 360.8 (165.3)**, ## | 91.5 (63.4) | 1228 (828)**, ##, $ | 3.8 (1.5)**, ##, $$, @ | 34.3 (27.3)##, @@ |
| Solv+GJ 24 hrs (I) | 21.9 (38.6) | 7.5 (10) | 23.1 (19.7) | 343.8 (193.6) | 112 (74) | n/a | 243.9 (414.9) | 1.5 (0.8)* | 6.54 (5.8) |
| Solv+GJ 48 hrs (D) | 44.9 (27.4) | 23.2 (14.4) | 70.8 (10.7) | 2296 (436.1)**, # | 632 (127.5)**, # | 56.2 (17.4) | 1889 (1026) | 19.22 (17.2) | 75.2 (118.8)@ |
| Solv+GJ 48 hrs (I) | 36.4 (50.1) | 36.9 (27.4) | 62.1 (22.0) | 1757 (1099) | 407.2 (299.4) | n/a | 2031 (1931) | 23 (20.2) | 339.2 (440.6)* |

Supplementary Table S1: BALF data for all measured cellular and non-cellular contents in minipigs receiving pulmonary treatments. Mean (±SD) shown. Statistically significant differences between groups: * group vs. sham control, # group vs. saline control, $ group vs. GJ, @ group vs. OP+GJ at similar time points. BALF: bronchoalveolar lavage fluid, WC: white cell, D: direct lung (right) injury, I: indirect lung (left) injury, n/a: not available. For the BALF IL-8, IL-6 and CRP indirect lung injury groups there was only n=4 for the solv+GJ group and the sham direct lung injury results were used for analysis. This was to maximize use of the ELISA plate when testing samples. NB: BALF samples from the indirectly-injured sham, and Solv +GJ, pig lungs at 48 hours had 98% and 97% RBC contamination, respectively, and may have been due to contamination by the biopsy sampling of the direct lung injury. These samples were removed from the total number of cells analysis but were included for a BALF white cell count (WCC) differential count and red blood cell count % BALF cells that were RBCs in a count of 100 cells, there were no significant differences. Modified from original thesis (1). Results shown as p value, <0.05*, p<0.01**, p<0.001***. Reproduced from original thesis (1).

| Group A | Group B | Combined or individual lung | P value | Significance |
| --- | --- | --- | --- | --- |
| Sham | OP+GJ | Combined lungs | 0.0003 | *** |
| Sham | Solv+GJ | Combined lungs | 0.16 | ns |
| Sham | GJ | Combined lungs | 0.0156 | * |
| Saline | OP+GJ | Combined lungs | 0.001 | ** |
| Saline | Solv+GJ | Combined lungs | 0.33 | ns |
| Saline | GJ | Combined lungs | 0.045 | * |
| OP+GJ | Solv+GJ | Combined lungs | 0.031 | * |
| OP+GJ | GJ | Combined lungs | 0.139 | ns |
| GJ | Solv+GJ | Combined lungs | 0.39 | ns |
| Sham | OP+GJ | Directly-injured lung | 0.005 | ** |
| Sham | Solv+GJ | Directly-injured lung | 0.06 | ns |
| Sham | GJ | Directly-injured lung | 0.0135 | * |
| Saline | OP+GJ | Directly-injured lung | 0.054 | ns |
| Saline | Solv+GJ | Directly-injured lung | 0.38 | ns |
| Saline | GJ | Directly-injured lung | 0.164 | ns |
| OP+GJ | Solv+GJ | Directly-injured lung | 0.38 | ns |
| OP+GJ | GJ | Directly-injured lung | 0.51 | ns |
| GJ | Solv+GJ | Directly-injured lung | 0.7 | ns |
| Sham | OP+GJ | Indirectly-injured lung | 0.037 | * |
| Sham | Solv+GJ | Indirectly-injured lung | 0.84 | ns |
| Sham | GJ | Indirectly-injured lung | 0.455 | ns |
| Saline | OP+GJ | Indirectly-injured lung | 0.0053 | ** |
| Saline | Solv+GJ | Indirectly-injured lung | 0.63 | ns |
| Saline | GJ | Indirectly-injured lung | 0.11 | ns |
| OP+GJ | Solv+GJ | Indirectly-injured lung | 0.0187 | * |
| OP+GJ | GJ | Indirectly-injured lung | 0.154 | ns |
| GJ | Solv+GJ | Indirectly-injured lung | 0.299 | ns |

**Supplementary Table S2: Histopathology lung score statistical analysis.** Table showing outcome of permutation tests (unpaired) of either combined (n=20), directly-injured (right, n=10) or indirectly-injured (left, n=10) lung histopathology scores between group A and group B. Note all comparisons of right and left lungs of the same group were non-significant when using unpaired permutation testing.

Results shown as p value, <0.05*, p<0.01**, p<0.001***. Tests between directly and indirectly-injured lungs of the same animals showed no significant difference. Reproduced from original thesis (1).

ns: non-significant.

**Supplementary Figures**


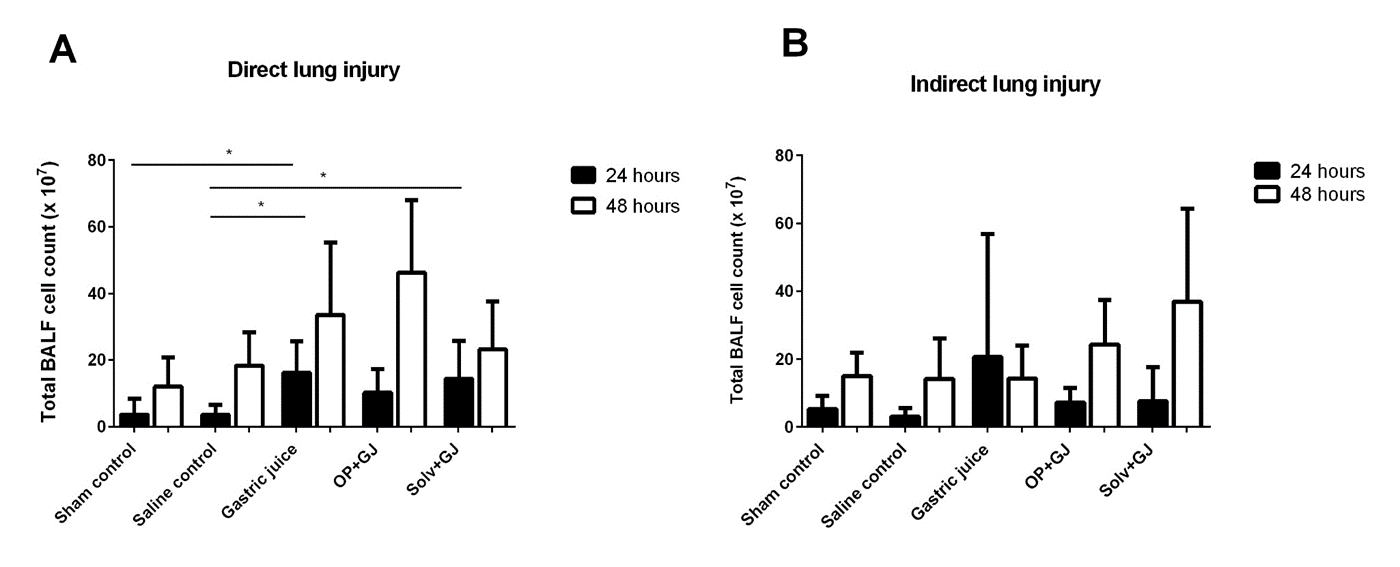


**Figure S1: Total BALF cell counts from the (A) directly-injured (right) and (B) indirectly-injured (left) lungs at 24 and 48 hours.** Kruskal-Wallis analysis of right or left lungs at 24 or 48 hours showed that only the difference between right lung groups at 24 hours (p=0.025) was significant. There was a significant difference between right and left lungs at 24 hours (p=0.03), but not 48 hours. Post hoc analysis of directly-injured (right) lung 24 hour groups using permutation tests are shown above. The graphs show mean and SD. Reproduced from original thesis (1).

NB:BALF samples from the indirectly-injured sham, and Solv+GJ, pig lungs at 48 hours had 98% and 97% RBC contamination, respectively, and may have been due to contamination by the bronchial biopsy sampling of the direct lung injury. These samples were removed from the total number of cells analysis, but were included for a BALF white cell count (WCC) differential count.


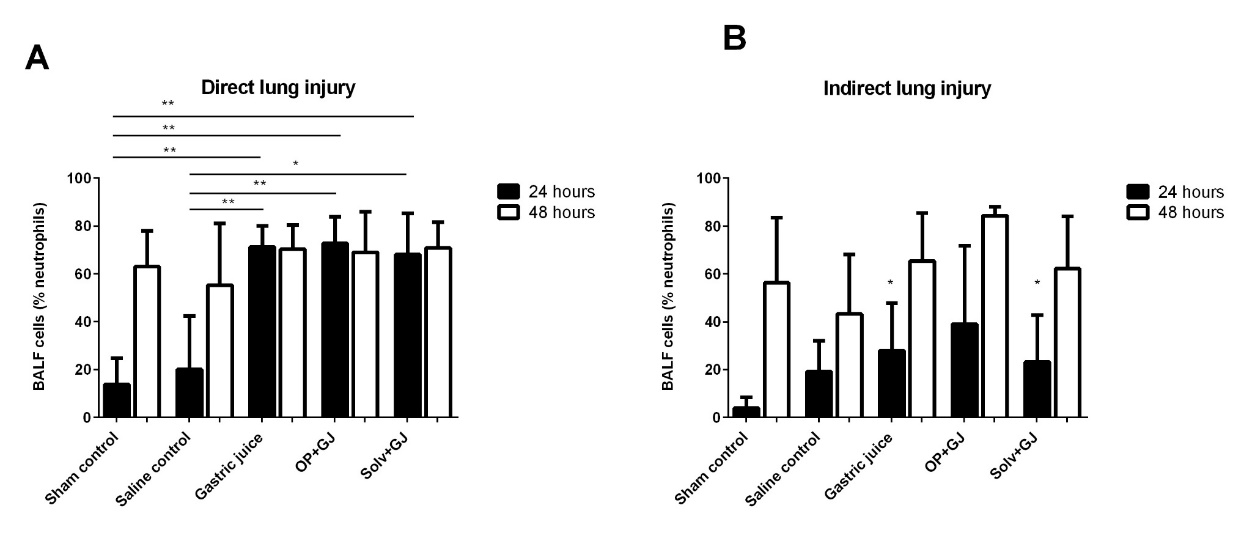


**Figure S2: Percentage of BALF white cells that are neutrophils from the (A) directly-injured (right) and (B) indirectly-injured (left) lungs at 24 and 48 hours.** Kruskal-Wallis analysis showed that only directly-injured lungs at 24 hours were significantly different (p= 0.002) between groups. There was a significant difference between right and left lungs at 24 hours (p= 0.0002) with post hoc permutation testing showing a significant difference between the GJ (p=0.016) and Solv + GJ (p=0.016) pig lungs, but not at 48 hours (shown by the asterisks and no bar). Data from one 48 hour indirectly-injured (left) lung from the Solv+GJ group were omitted due to a lost cytospin slide. Post hoc analysis using permutation tests are shown above. Graphs show means and SD. Reproduced from original thesis (1).


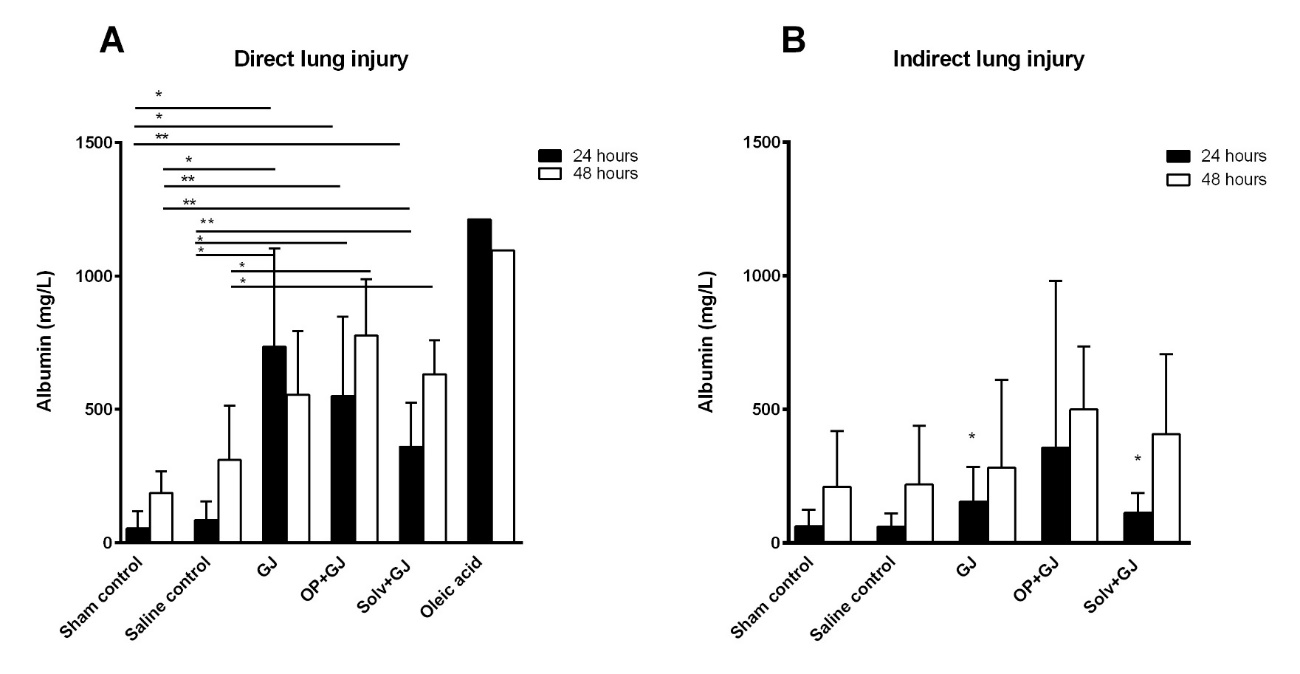


Figure S3: Albumin concentrations in the BALF of minipigs receiving pulmonary treatments. Albumin in BALF from (A) directly-injured (right) and (B) indirectly-injured (left) lungs at 24 and 48 hours. Kruskal-Wallis analysis of right lungs of all groups at 24 (p=0.0032) or 48 (p=0.0041) hours was significant between groups (n=5), but not for left lungs. Right and left lungs at 24 (p=0.0018) and 48 (p= 0.0085) hours had a significant difference, post hoc tests (asterisks no bars) showed GJ (p=0.0238) and Solv+GJ (p=0.0159) lungs showed a difference at 24 hours, but not at 48 hours. Post hoc analysis using permutation tests detailed as above. Mean and SD shown. Reproduced from original thesis.


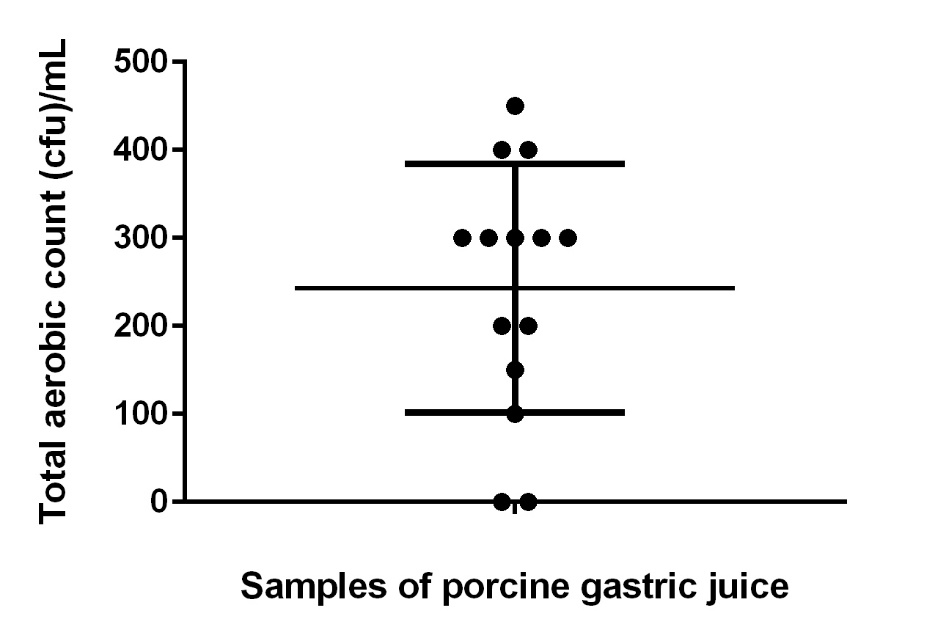


**Figure S4: Dot plot showing bacterial growth from porcine gastric juice aliquots (n=14) used in the aspiration study.** Fourteen GJ samples were sent for aerobic bacterial culture and total aerobic count (TAC) reported as colony forming units (cfu)/mL. The TAC of the porcine GJ was 243±141cfu/mL (mean±SD). The predominant organism was the *Bacillus* species and was morphologically different to the bacteria seen in the BALF specimens at 48 hours. Therefore, the GJ itself was unlikely to be the source of high TAC in the aspiration study groups at 48 hours. Mean and SD shown. Reproduced from original thesis (1).





**Figure S5:** **Bacterial numbers in cultured pooled (n=24) tracheal aspirates at -30 min and 48h BALF samples.** Number of aerobic bacteria (cfu/mL) in -30 min pooled (n=24) tracheal aspirates and 48-hour BALF samples. There was no significant difference between 48 hours BALF samples. Mean and SD are shown.

**References**

1. Hulse E. Respiratory Complications of Organophosphorus Pesticide Poisoning. University of Edinburgh; 2016. p. p1-401.
